# Evaluating the pro-survival potential of apoptotic bodies derived from 2D- and 3D- cultured adipose stem cells in ischaemic flaps

**DOI:** 10.1186/s12951-024-02533-1

**Published:** 2024-06-14

**Authors:** Gaoxiang Yu, Jian Ding, Ningning Yang, Lu Ge, Nuo Chen, Xuzi Zhang, Qiuchen Wang, Xian Liu, Xuanlong Zhang, Xiaoqiong Jiang, Yibo Geng, Chenxi Zhang, Jiadong Pan, Xiangyang Wang, Weiyang Gao, Zhijie Li, Hongyu Zhang, Wenfei Ni, Jian Xiao, Kailiang Zhou, Liangliang Yang

**Affiliations:** 1 Department of Orthopaedics, The Second Affiliated Hospital and Yuying Children’s Hospital of Wenzhou Medical University, Wenzhou, 325027 China; 2 School of Pharmaceutical Sciences, Cixi Biomedical Research Institute, Wenzhou Medical University, Wenzhou, 325035 China; 3 Zhejiang Provincial Key Laboratory of Orthopaedics, Wenzhou, 325027 China; 4 The Second Clinical Medical College of Wenzhou Medical University, Wenzhou, 325027 China; 5 Department of Hand Surgery, Ningbo Sixth Hospital, Ningbo, 315042 China

**Keywords:** Adipose-derived mesenchymal stem cells, 3D cell culture, Apoptotic bodies, MicroRNAs, Ischaemic flap

## Abstract

In the realm of large-area trauma flap transplantation, averting ischaemic necrosis emerges as a pivotal concern. Several key mechanisms, including the promotion of angiogenesis, the inhibition of oxidative stress, the suppression of cell death, and the mitigation of inflammation, are crucial for enhancing skin flap survival. Apoptotic bodies (ABs), arising from cell apoptosis, have recently emerged as significant contributors to these functions. This study engineered three-dimensional (3D)-ABs using tissue-like mouse adipose-derived stem cells (mADSCs) cultured in a 3D environment to compare their superior biological effects against 2D-ABs in bolstering skin flap survival. The findings reveal that 3D-ABs (85.74 ± 4.51) % outperform 2D-ABs (76.48 ± 5.04) % in enhancing the survival rate of ischaemic skin flaps (60.45 ± 8.95) % (all *p* < 0.05). Mechanistically, they stimulated angiogenesis, mitigated oxidative stress, suppressed apoptosis, and facilitated the transition of macrophages from M1 to M2 polarization (all *p* < 0.05). A comparative analysis of microRNA (miRNA) profiles in 3D- and 2D-ABs identified several specific miRNAs (miR-423-5p-up, miR30b-5p-down, etc.) with pertinent roles. In summary, ABs derived from mADSCs cultured in a 3D spheroid-like arrangement exhibit heightened biological activity compared to those from 2D-cultured mADSCs and are more effective in promoting ischaemic skin flap survival. These effects are attributed to their influence on specific miRNAs.

## Introduction

In trauma surgery, the primary objective is to repair and reconstruct damaged tissues to restore normal physiological barriers (skin flap) [1]. Flap transplantation is the surgical method used to restore these barriers [2, 3]. Necrosis of the transplanted flap indicates operation failure [4, 5]. The main cause of flap necrosis is ischaemia, especially prevalent in areas with insufficient blood supply, such as the distal end of the flap [6, 7]. With the ischaemic necrosis of the flap, patients need to undergo a second operation, leading to secondary physical and psychological pressure [8]. Currently, the management of ischaemic skin flaps predominantly revolves around promoting angiogenesis, preventing cell death, mitigating oxidative stress, and suppressing inflammatory responses [9–12]. Consequently, identifying an effective treatment strategy to address these concerns and rescue the ischaemic flap is crucial.

In recent years, researchers have discovered the miraculous potential of stem cells to differentiate into various types of cells in skin tissue, bringing new hope for the treatment of ischaemic flaps [13–15]. A large body of evidence indicates that adipose-derived stem cells (ADSCs) transplantation can promote angiogenesis, inhibit cell death, mitigate oxidative stress, and suppressing inflammatory of wound healing [13, 16–19]. A clinical trial investigated the use of ADSC-enriched fat grafts for the treatment of chronic ulcers in patients with peripheral arterial disease. The results demonstrated significant improvements in wound healing and vascularization, highlighting the therapeutic potential of ADSCs in promoting tissue repair and regeneration in ischemic conditions [20]. Consequently, mADSCs was selected as the subject of investigation regarding ischaemic skin flaps.

Studies have shown that although stem cell transplantation therapy has certain efficacy in treating ischaemic flap, the safety of its source, ethics, and poor retention and differentiation capabilities after implantation limit the application of exogenous transplanted stem cells [21]. Extracting extracellular vesicle (EVs) from stem cells for therapy has been widely studied [22]. Recent research findings that stem cells injected into the body will quickly undergo apoptosis and generate apoptotic bodies (ABs), which are the main way that stem cells exert their therapeutic effects [23]. Therefore, extracting ABs from ADSCs rather than directly transplanting cells may provide better results. These results imply that the generation of ABs rather than cell differentiation may be the cause of ADSCs’ therapeutic impact on flaps. ABs, a type of EVs in the size range of 0.5–5 μm, are generated during apoptosis and mediate intercellular exchange of bioactive molecules [23, 24]. Their composition primarily reflects the molecular properties of their parent cell, indicating that changes in the microenvironment of these cells can influence the protein and micro-RNA (miRNA) content of ABs [25]. Micro RNAs are small non-coding RNAs of 20∼25 nucleotides that regulate protein production through targeted manipulation of mRNA [26, 27]. A growing body of literature emphasizes the regulatory potential of miRNAs in diverse cellular processes including angiogenesis, oxidative stress, cell death, macrophage polarization and ischaemic flap therapy [26–29]. Notably, ABs harbor specific miRNA profiles inherited from their parent cells [25, 30, 31]. Therefore, the possibility of identifying ABs extracted from appropriate maternal cells for improving the treatment of ischemic flaps is feasible.

Following hypoxic stimulation, circ-Snhg11 experiences upregulation within ADSCs-derived exosomes. This upregulation promotes angiogenesis and expedites the healing of diabetic wounds [32, 33]. Additionally, inflammation-stimulated MSCs-derived EVs regulate macrophages through the targeting of CSF-1 via the delivery of miR-27b-3p, further facilitating temporomandibular joint condylar regeneration [34]. ABs sourced from different microenvironments exert specific therapeutic effects due to cargo specificity [25]. This illustrates our capacity to modulate the growth of ADSCs, thereby enhancing their biological activity and resulting in ABs with enhanced therapeutic efficacy.

Natural tissues and organs exhibit direct cell-cell and cell-matrix interactions in three-dimensional (3D) structures not replicable in traditional 2D cell culture, which play critical roles in regulating stem cell behavior, differentiation, and function [35–37]. Moreover, 3D culture provides a more physiologically relevant environment by simulating key aspects of the in *vivo* niche, including nutrient gradients, oxygen tension, and mechanical forces [38]. These factors influence stem cell fate decisions and functional properties, such as proliferation, differentiation, and paracrine signaling [39]. By better recapitulating these physiological cues, 3D-cultured pheroids ADSCs are hypothesized to exhibit enhanced biological activity compared to 2D cultures. Overall, 3D cultured pheroids ADSCs are believed to exhibit superior biological activity than 2D cultured ADSCs. Currently, no studies investigate the effectiveness of ABs derived from 3D-cultured ADSCs for treating ischaemic skin flaps. Hence, we conjecture with confidence that 3D-cultured pheroids ADSCs exhibit superior biological activity compared to their 2D counterparts, and the ABs derived from them yield more pronounced biological activity-enhancing effects. We also acknowledge the potential limitations and challenges associated with using ABs in the treatment of ischeamic flaps, including issues related to clearance and immunogenicity, dose optimization, targeting and specificity, mechanistic understanding, and clinical translation.

**Scheme 1:**
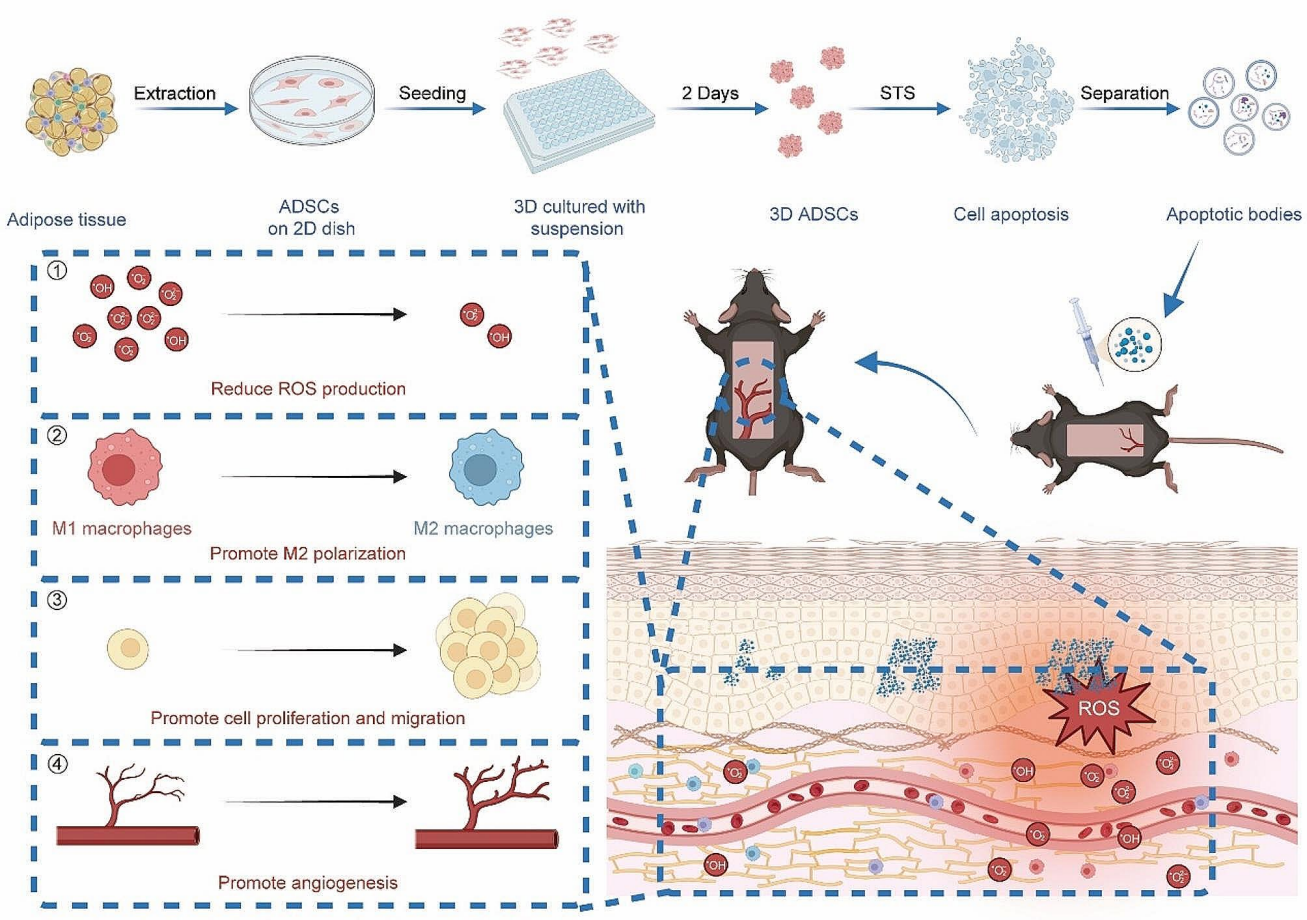
Schematic illustration of 3D-ABs improves the viability of ischaemic flaps by stimulating angiogenesis, suppressing oxidative stress, and enhancing M1 to M2 polarization in macrophages

In this study, mouse adipose-derived stem cells (mADSCs) were cultured both in basic cell culture dishes and cell spheroid culture wells to generate mADSCs in 2D and 3D states, respectively (Scheme 1). Apoptosis is induced using chemical methods (Streptothricin; STS) to extract ABs. Additionally, the potential effects of these ABs on reducing ROS production, and promoting macrophage M2 polarization, cell proliferation and migration and angiogenesis were investigated both in *vitro* and in *vivo*(Scheme 1). We conducted a detailed investigation into the distinctions in ABs between mADSCs cultured in 2D and those cultured in 3D using miRNA sequencing analysis. Our findings clarify key characteristics of ABs derived from mADSCs and provide valuable insights to support the clinical use of ABs in managing ischaemic flap necrosis.

## Results

### Isolation and characterization of 2D and 3D-ABs

**Fig. 1:**
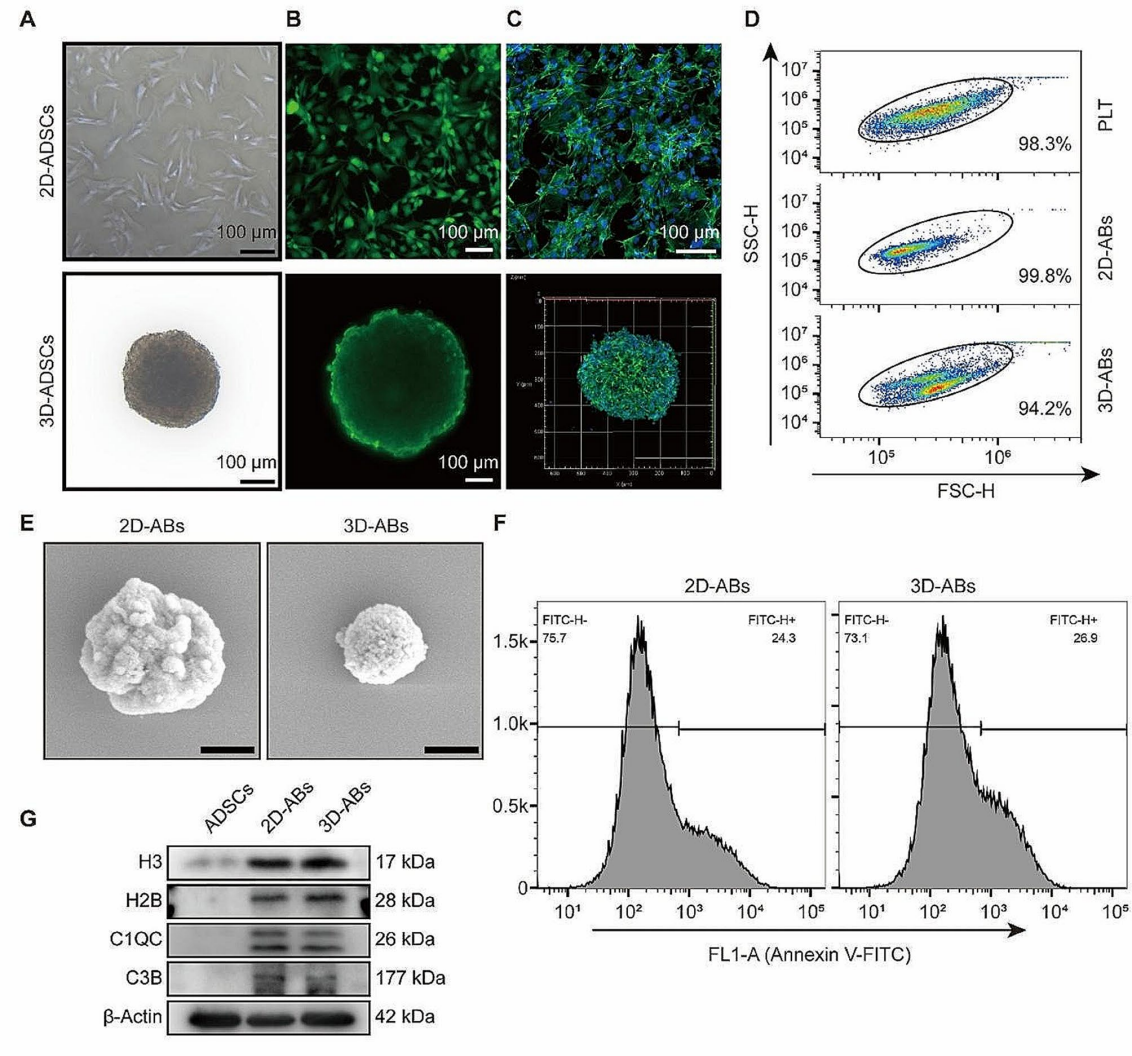
Isolation and characterization of 2D and 3D-ABs. (**A**) The morphology of 2D and 3D-mADSCs. (**B**) Representative live/ dead images of 2D and 3D-mADSCs. (**C**) F-actin immunofluorescence of 2D and 3D-mADSCs (nuclei: hoechst 33,342). (**D**) FSC/SSC analysis of 2D and 3D-ABs. Frames were used as size marker (platelet, PLA, 1–4 μm) gating 2D and 3D-ABs. (**E**) SEM micrographs of 2D and 3D-ABs. Scale bar, 0.5 μm. (**F**) Using annexin V-FITC staining, FCM analysis was used to identify the 2D and 3D-ABs (*n* = 3). (**G**) WB analysis of C1QC, H3, C1QC, C3B, H2B and β-Actin in the indicated groups

The study employed a suspension method to generate 3D spherical adipose-derived stem cells (3D-mADSCs) from 2D-mADSCs. The biocompatibility of the 2D and 3D cellular structures was assessed through microscopy, live/dead experiments, and FITC-Phalloidin (F-actin) staining **(**Fig. 1A-C**)**. The figure illustrates the successful preparation of 3D-mADSCs and their robust survival.

Next, 2D- and 3D-ABs were isolated from mADSCs cultured on conventional cell culture dishes and 3D spheres using standard ultracentrifugation techniques [40]. The size distribution of 2D and 3D-ABs, primarily determined by flow cytometry (FCM) analysis utilizing FSC/SSC, was observed to predominantly fall within the size range comparable to that of platelets (utilized as the reference standard) **(**Fig. 1D**)** [25]. The morphology of 2D and 3D-ABs was observed through scanning electron microscopy (SEM), revealing their spherical vesicular structure with an approximate size of 1 μm **(**Fig. 1E**)**. Phosphatidylserine serves (PS) as a distinctive surface marker of apoptotic cells, and ABs resulting from apoptosis exhibit unique PS expression that sets them apart from other EVs [41]. In order to assess the purity of 2D and 3D-ABs, Annexin-V/FITC staining and FCM analysis were conducted to identify the purified ABs. The FCM results showed that the extracted 2D-ABs and 3D-ABs positive for phosphatidylserine (PS) **(**Fig. 1F**)**. Western blotting (WB) was performed and demonstrated that the ABs markers H3, H2B, C1QC, and C3B had positive expression in the samples **(**Fig. 1G**)**. In summary, 3D-mADSCs were successfully prepared, and 3D-ABs were extracted.

### 3D-ABs promoted the survival and inhibited oxidative stress and apoptosis of Hypo-HUVECs

**Fig. 2:**
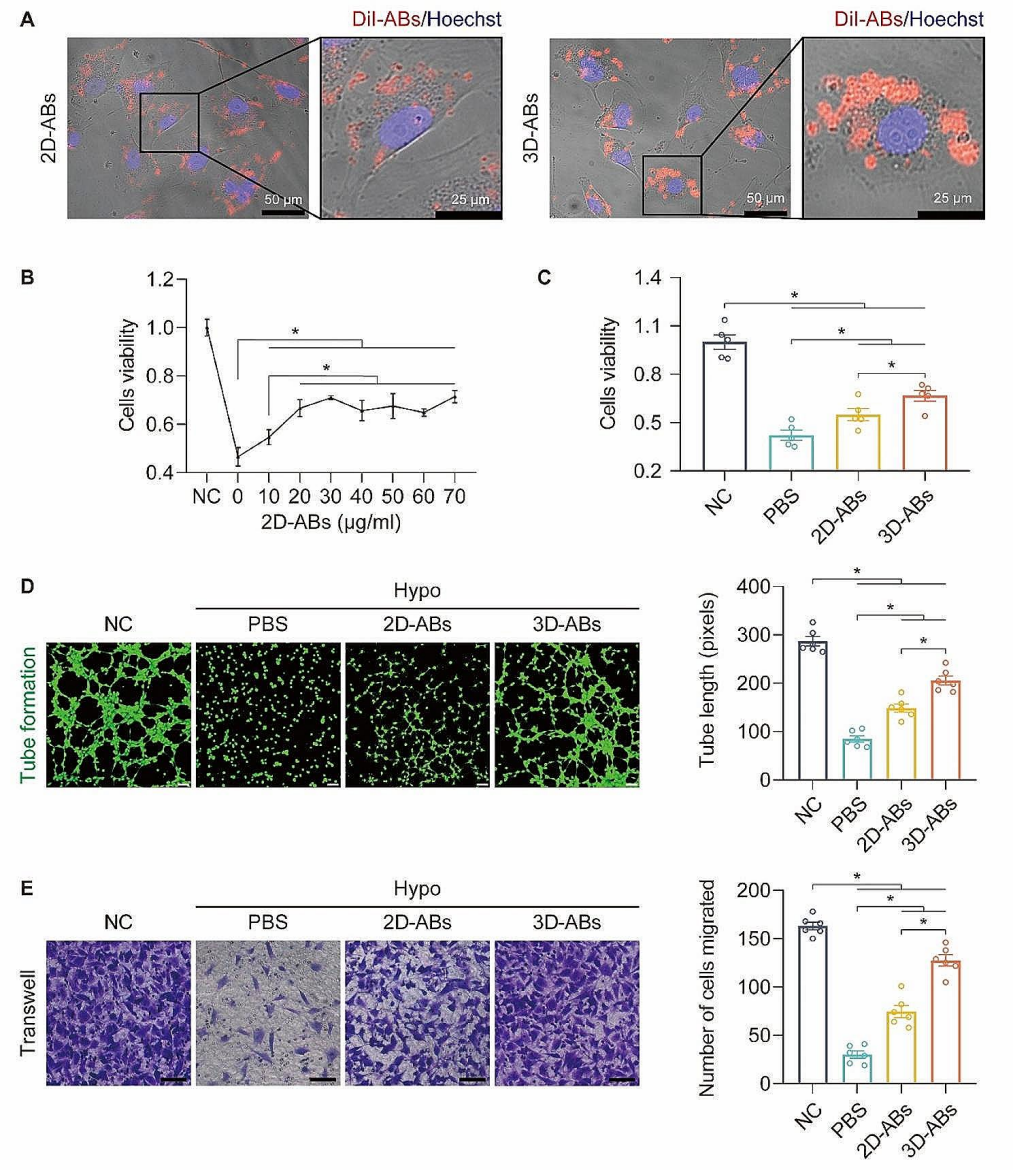
3D-ABs promoted the survival of HUVECs. (**A**) Uptake of 2D and 3D-ABs in HUVECs detected by confocal microscopy. (**B**) Proliferation of Hypo-HUVECs after 2D-ABs treatment measured by CCK-8 assays. (**C**) Proliferation of Hypo-HUVECs after 2D and 3D-ABs treatment measured by CCK-8 assays. (**D**) After treatment for 24 h, Hypo-HUVECs were divided into four groups and exposed to an in *vitro* angiogenesis (tube formation) experiment. The findings showed that the cells could be cultured for six hours. Scale bars, 100 μm. Quantification of tube length (pixels) among the four groups (*n* = 6). (**E**) After treatment for 24 h in each of the four groups, Hypo-HUVECs were subjected to cell migration experiments; the data shown here were obtained after 12 h of culture. Scale bars, 100 μm. Quantification and analysis of the number of cells migrating throughout a 12-hour period in each of the four groups (*n* = 6). SEM error bars are used. Significance (*): p value < 0.05; equal variances ANOVA with LSD post hoc analysis or unequal variances Dunnett’s T3 technique

Promoting the survival and proliferation of endothelial cells is critical for the survival of ischaemic flaps, as the distance that new blood vessels can reach determines the survival area of the flap [42]. In this work, the therapeutic effects of 2D and 3D-ABs were examined using human umbilical vein endothelial cells (HUVECs). Confocal microscopy showed that almost all HUVECs engulfed DiI-labeled ABs **(**Fig. 2A**)**.

First, different concentrations of 2D-ABs were used to treat hypoxia-damaged HUVECs (Hypo-HUVECs). As the concentration of 2D-ABs approached 20 µg/ml, the CCK-8 data revealed no discernible change in the therapeutic effect **(**Fig. 2B**)**. The therapeutic effects of 2D-ABs and 3D-ABs were compared, and a consistent concentration of 10 µg/ml was chosen. The CCK-8 results showed that, under the same concentration, 3D-ABs had a better effect on promoting the survival of Hypo-HUVECs compared to 2D-ABs **(**Fig. 2C**)**. Then, the tube formation assay of HUVECs showed that 3D-ABs had better ability to promote the tube formation of Hypo-HUVECs compared to 2D-ABs **(**Fig. 2D**)**. Moreover, the transwell experiments demonstrated that 3D-ABs-treated Hypo-HUVECs exhibited stronger migratory ability than those treated with 2D-ABs **(**Fig. 2E**)**.

**Fig. 3:**
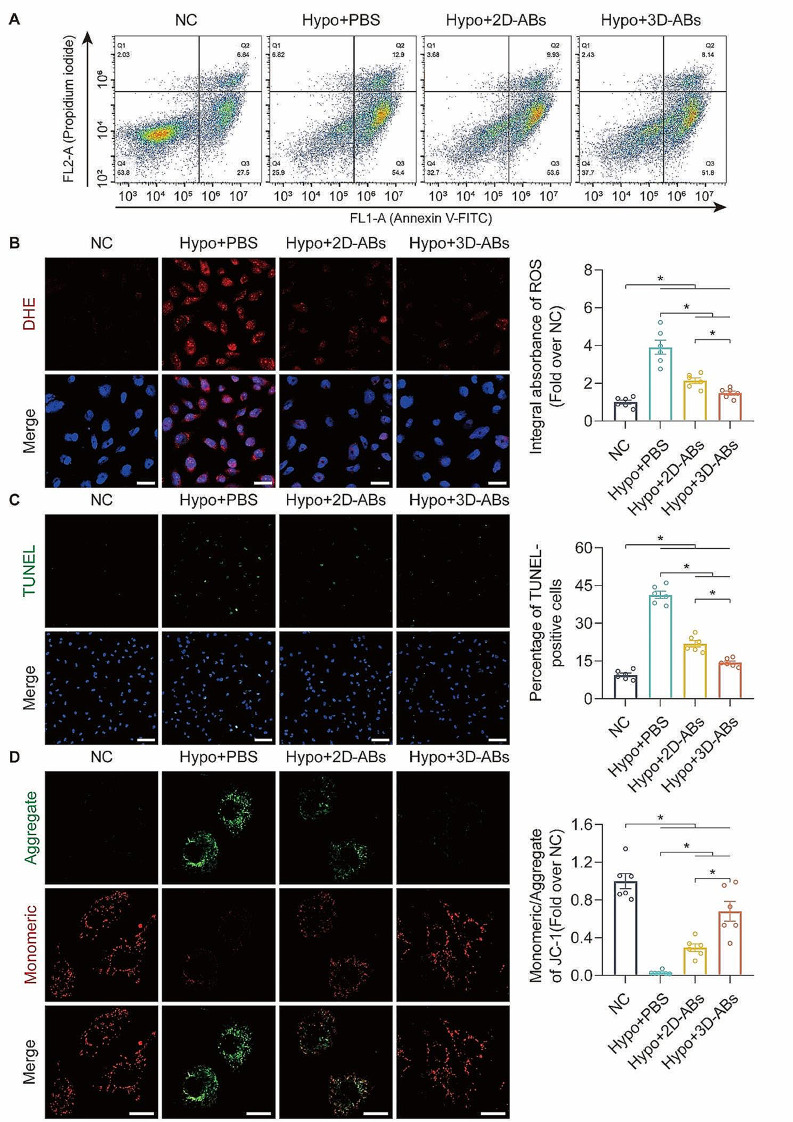
3D-ABs inhibited oxidative stress and apoptosis of HUVECs. (**A**) Hypo-HUVECs treated after 24 h among the four groups determined by FCM analysis using annexin V and PI staining (*n* = 3). (**B**) DHE staining to detect ROS damage in Hypo-HUVECs among the four groups (nuclei: hoechst 33,342). Scale bars, 50 μm. Analysis and quantification of DHE integral absorbance in each of the four groups (*n* = 6). (**C**) TUNEL staining to detect cell death of Hypo-HUVECs among the four groups (nuclei: hoechst 33,342). Scale bars, 100 μm. Analysis and quantification of TUNEL-positive HUVECs among the four groups (*n* = 6). (**D**) Representative images of JC-1 staining in Hypo-HUVECs among the four groups after treatment for 24 h. Scale bars, 20 μm. Quantification of the ratio of monomeric/aggregated JC-1 among the four groups after treatment for 24 h (*n* = 6). SEM error bars are used. Significance (*): p value < 0.05; equal variances ANOVA with LSD post hoc analysis or unequal variances Dunnett’s T3 technique

We then investigated the ability of ABs to resist apoptosis and hypoxic injury HUVECs. The results of Annexin V/PI staining showed that 3D-ABs demonstrated a stronger anti-apoptotic ability in Hypo-HUVECs **(**Fig. 3A**)**. The DHE staining indicated that 3D-ABs had better performance in removing oxidative damage compared to 2D-ABs **(**Fig. 3B**)**. Moreover, the TUNEL staining demonstrated that treatment with 3D-ABs could reduce cell death **(**Fig. 3C**)**. Finally, JC-1 staining revealed that 3D-ABs had a stronger ability to recover mitochondrial membrane potential compared to 2D-ABs **(**Fig. 3D**)**. In summary, when treating Hypo-HUVECs, 3D-ABs have stronger abilities in promoting cell viability, inhibiting cell apoptosis, and reducing cellular oxidative damage compared to 2D-ABs.

### 3D-ABs promoted the M1 to M2 transition of RAW 264.7

**Fig. 4:**
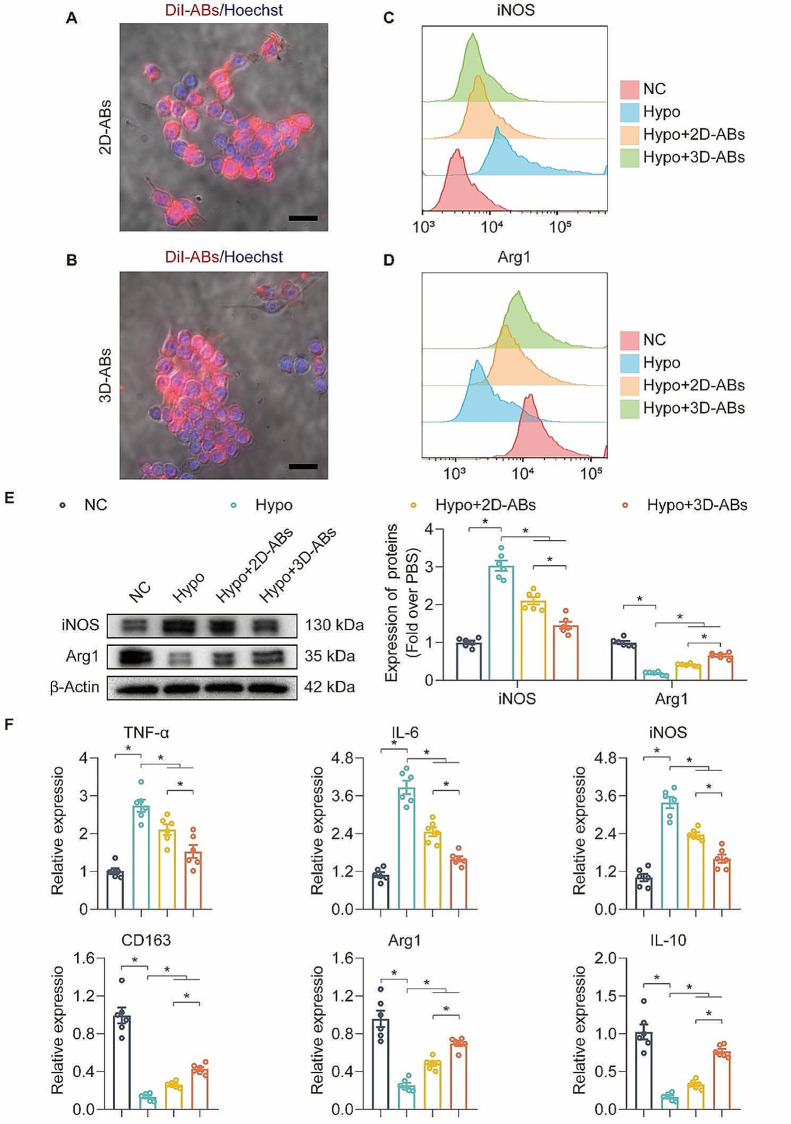
3D-ABs promoted the M1 to M2 polarization in macrophages in *vitro*. (**A**) The uptake of 2D-ABs was seen in RAW 264.7 cells using confocal microscopy. (**B**) The uptake of 3D-ABs was seen in RAW 264.7 cells using confocal microscopy. (**C**) After treatment for 24 h (*n* = 3), FCM analysis of M1 (iNOS) Raw264.7 cells was performed across the 4 groups. (**D**) After treatment for 24 h (*n* = 3), FCM analysis of M2 (Arg1) Raw264.7 cells was performed across the 4 groups. (**E**) After treatment for 24 h, WB analysis of iNOS and Arg1 proteins level in RAW264.7 cells was performed across the 4 groups. Quantified proteins level among the four groups. β-Actin served as a loading control and for band density normalization (*n* = 6) **(F)** Comparison of the relative TNF-α, IL-6, iNOS, CD163, Arg1 and IL-10 expression levels in RAW264.7 cells among the four groups after treatment for 24 h (*n* = 6). SEM error bars are used. Significance (*): p value < 0.05; equal variances ANOVA with LSD post hoc analysis or unequal variances Dunnett’s T3 technique

In the process of promoting the survival of ischaemic flaps, anti-inflammatory effects are also worth considering [43, 44]. RAW 264.7 cells (mouse macrophages) were used in this study, and confocal microscopy showed that DiI-ABs were internalized by RAW 264.7 cells **(**Fig. 4A-B**)**. Furthermore, in the context of hypoxic injury, macrophages tended to polarize towards the M1 phenotype, while ABs promoted RAW 264.7 cells to polarize from M1 to M2 phenotype, and 3D-ABs exhibited stronger ability to promote M2 polarization compared to 2D-ABs **(**Fig. 4C-D**)**. Western blotting (WB) results showed that iNOS protein decreased and Arg1 protein increased in Hypo-RAW264.7 cells after ABs treatment, and the therapeutic effect of 3D-ABs was stronger than that of 2D-ABs **(**Fig. 4E**)**. And qPCR detected M1 (TNF-α, IL-6 and iNOS) and M2 (CD163, Arg1 and IL-10)-related gene mRNA. The results showed that the addition of ABs in Hypo-RWA264.7 cells reduced the expression of M1-related genes and increased the expression of M2-related genes, among which 3D-ABs showed a stronger effect. Overall, ABs promoted the polarization of Hypo-macrophages from M1 to M2 in *vitro*, and 3D-ABs show a stronger effect.

### 3D-ABs promoted survival, stimulated angiogenesis and suppressed oxidative stress in ischaemic flaps

**Fig. 5:**
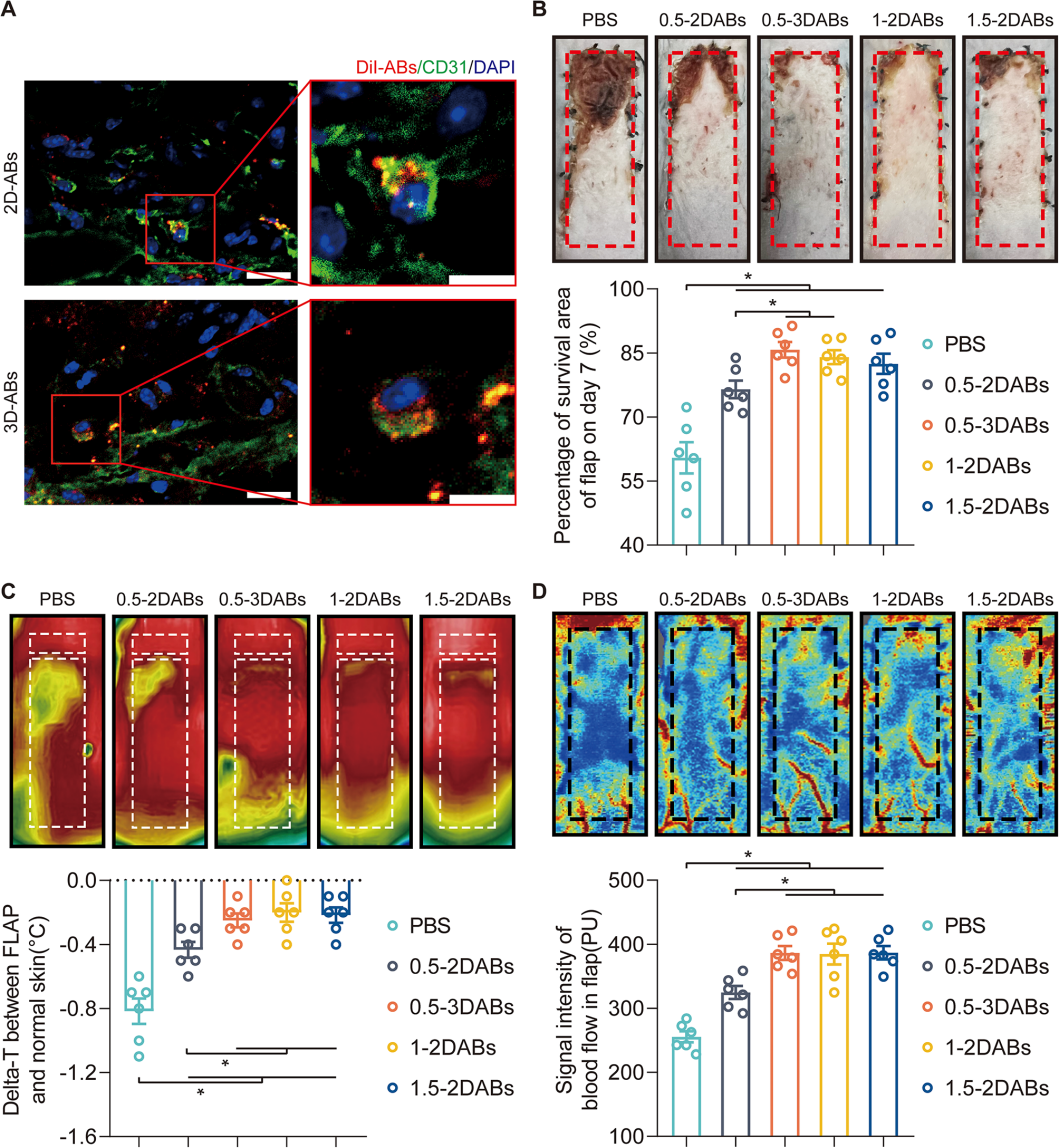
3D-ABs promoted the survival of ischaemic flaps. (**A**) Uptake of 2D and 3D-ABs in CD31-positive cells on POD3 detected by confocal microscopy. Left scale bar: 20 μm; right scale bar: 10 μm. (**B**) Digital photograph of the flap survival area on POD7. Quantified percentage of survival area among the five groups on POD7 (*n* = 6). (**C**) Thermal images of the flap on POD7. Comparison of the Delta-T between the flap and normal skin among the five groups on POD7. (**D**) Images of the subcutaneous blood flow network on POD7. Quantified blood flow signal intensity in ischaemic flaps among the five groups on POD7 (*n* = 6). SEM error bars are used. Significance (*): p value < 0.05; equal variances ANOVA with LSD post hoc analysis or unequal variances Dunnett’s T3 technique

In *vivo* study, DiI-labeled ABs were injected in skin flap during the surgery, and on postoperative day (POD) 3, CD31-positive endothelial cells (ECs) internalized DiI-ABs **(**Fig. 5A**)**. To further evaluate their therapeutic efficacy, 2D and 3D-ABs were injected into the skin flap. On POD7, both 2D and 3D-ABs showed the ability to promote skin flap survival **(**Fig. 5B**)**. The therapeutic effect of 2D-ABs at concentrations of 1 and 1.5 mg/ml was stronger than that of 0.5 mg/ml **(**Fig. 5B**)**. Interestingly, under the same concentration, 3D-ABs had a stronger therapeutic effect on promoting flap survival than 2D-ABs **(**Fig. 5B**)**. The next step was to evaluate the survival quality of the flap. To eliminate the individual temperature difference, we calculated Δ-T to represent the survival quality of the flap by subtracting the temperature of the normal skin at the head end from the average temperature of the upper part of the flap area **(**Fig. 5C**)**. The lower the Δ-T, the lower the survival quality of the flap, and Δ-T is close to zero in the high survival quality flap. The statistical results showed that ABs could improve the survival quality of the ischaemic flap, and 3D-ABs had a better therapeutic effect than 2D-ABs at the same concentration **(**Fig. 5C**)**. Subsequently, we employed laser Doppler flowmetry to examine the subcutaneous vascular network of the skin flap, and found that ABs could strengthen the intensity of the blood flow signal in ischaemic skin flaps **(**Fig. 5D**)**. Notably, 3D-ABs exhibited a more potent ability to promote blood flow signals than 2D-ABs at the same concentration.

**Fig. 6:**
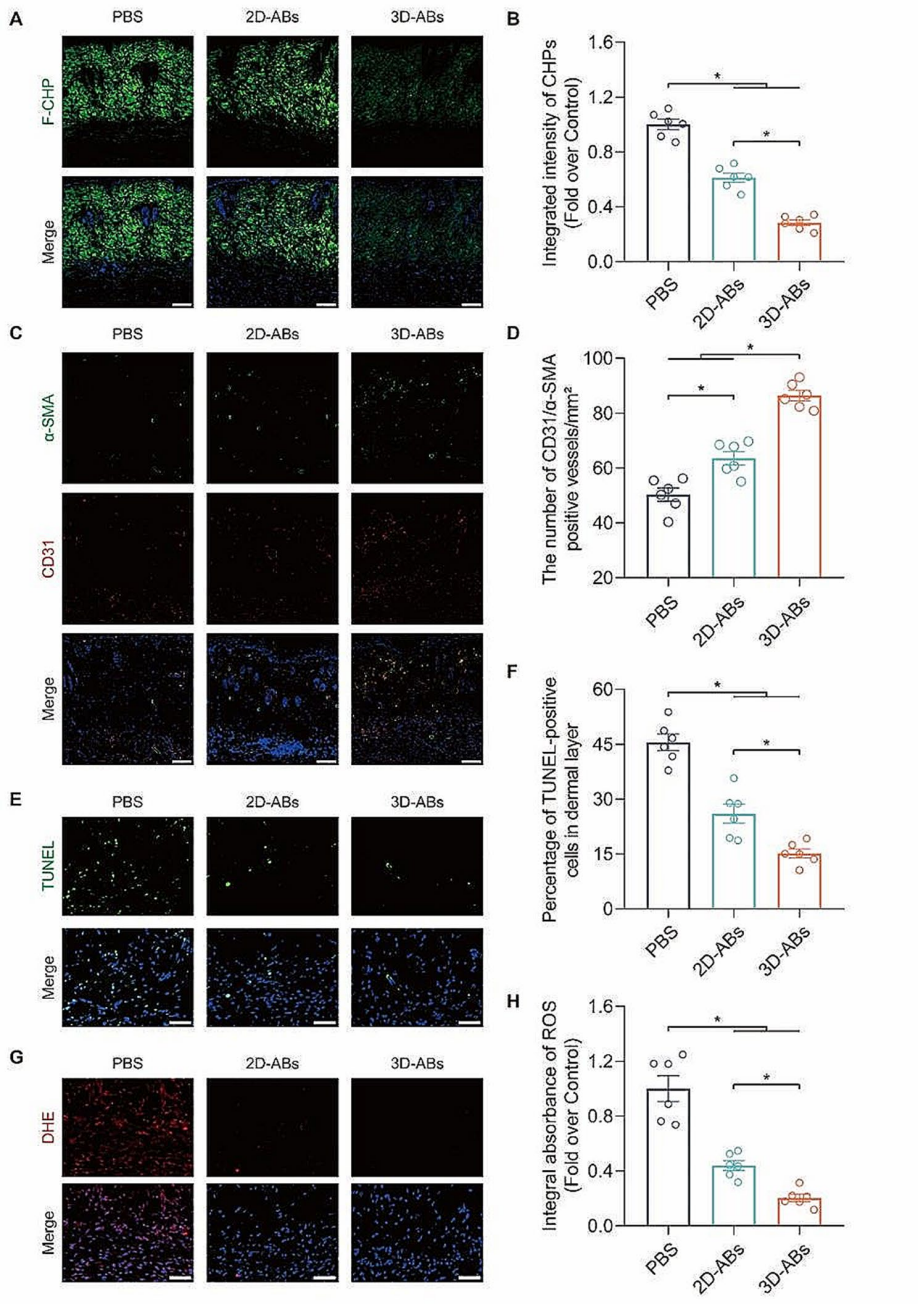
3D-ABs suppress oxidative stress and cell death, and stimulated angiogenesis in ischaemic flaps. (**A**) F-CHP staining for damaged collagen detection in the skin on POD7. Scale bars, 100 μm. (**B**) The three groups’ quantified F-CHP intensity (*n* = 6). (**C**) CD31 and α-SMA IF staining in the flap on POD7. Scale bars, 50 μm. (**D**) Quantified CD31/α-SMA-positive blood vessel density among the three groups (*n* = 6). (**E**) Dead cells in flap tissue sections on POD7 under the detection of TUNEL staining. Scale bar, 20 μm. (**F**) TUNEL-positive cell percentage in the dermal layer, quantified for each of the three groups (*n* = 6). (**G**) DHE staining of the skin tissues on POD7 among the three groups. Scale bars, 50 μm. (**H**) Quantified DHE among the three groups (*n* = 6). SEM error bars are used. Significance (*): p value < 0.05; equal variances ANOVA with LSD post hoc analysis or unequal variances Dunnett’s T3 technique

Based on the aboving results, we conducted our experiment in *vivo* using PBS as the control group, and 0.5 mg/ml concentration of 2D and 3D-ABs as the experimental groups. We took the surviving-necrotic border flap tissue for further investigation. The F-CHP staining results indicated that ABs reduced collagen necrosis in ischaemic flaps, and the 3D-ABs group exhibited better therapeutic effects than the 2D-ABs group **(**Fig. 6A-B**)**. The investigation of angiogenesis showed a notable rise in blood vessels positive for CD31/α-SMA upon the application of ABs, with 3D-ABs exhibiting a better ability to promote angiogenesis than 2D-ABs. The results of immunofluorescence (IF) demonstrated an important rise in the quantity of blood vessels positive for CD31/α-SMA with the application of ABs, and 3D-ABs exhibited a better pro-angiogenic effect compared to 2D-ABs **(**Fig. 6C-D**)**. According to the TUNEL staining results, ABs were found to significantly reduce cell death in the tissue, with 3D-ABs demonstrating greater efficacy than 2D-ABs in this regard **(**Fig. 6E-F**)**. The DHE staining results showed that ABs effectively reduced ROS accumulation in the flap tissue, and the level of ROS was lower in the 3D-ABs group compared to the 2D-ABs group **(**Fig. 6G-H**)**. In conclusion, ABs have good effects on promoting survival of ischaemic flaps, enhancing angiogenesis, and inhibiting oxidative stress. Moreover, 3D-ABs exhibit better therapeutic effects compared to 2D-ABs for ischaemic flaps.

### 3D-ABs enhanced M1 to M2 polarization in ischaemic flaps

**Fig. 7:**
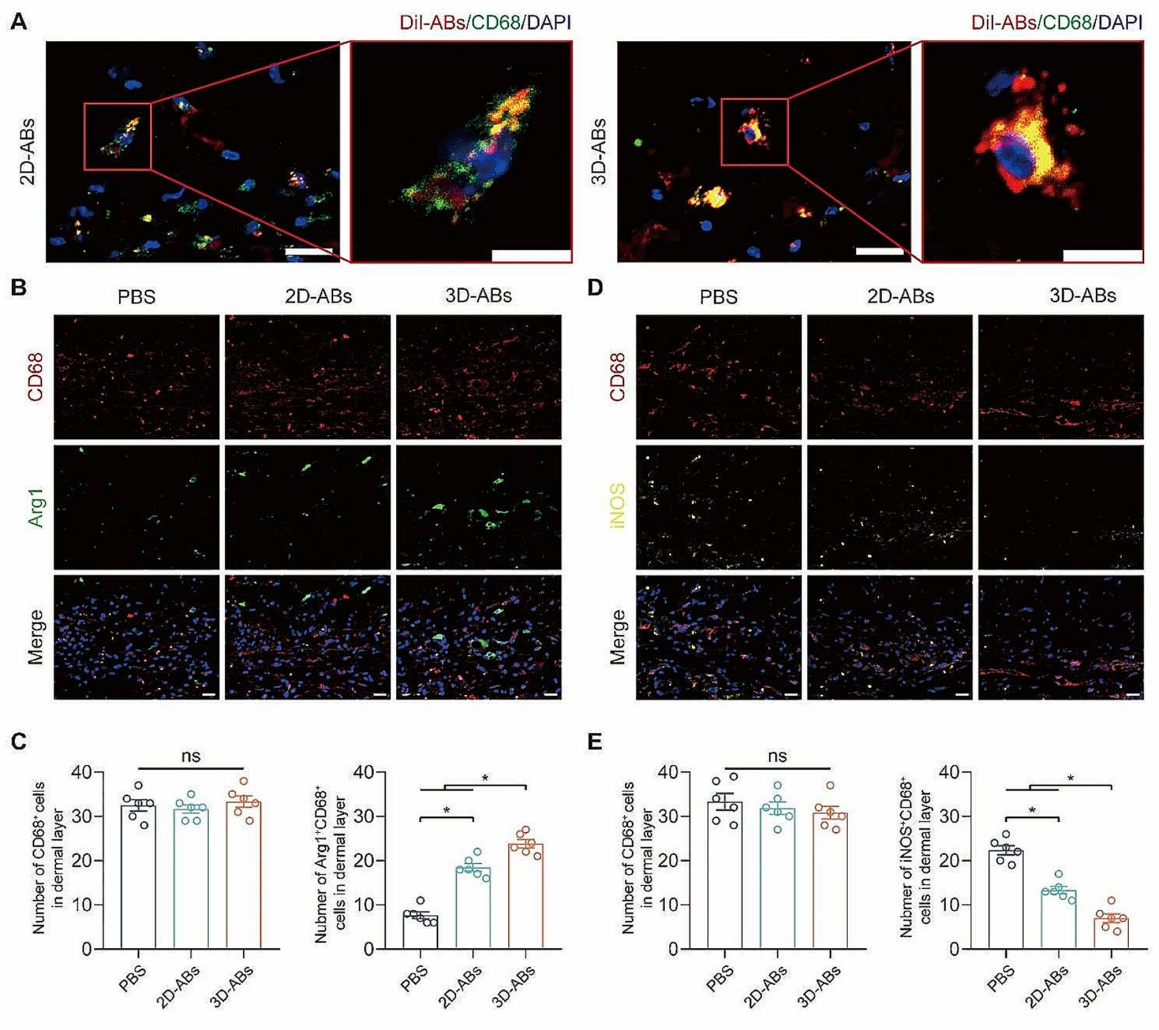
3D-ABs enhanced M1 to M2 polarization in macrophages in ischaemic flaps. (**A**) Uptake of 2D and 3D-ABs in CD68-positive cells on POD3 detected by confocal microscopy. Scale bars, Left scale bar: 20 μm; right scale bar: 10 μm. (**B**) CD68 and Arg1 staining CD68 in the flap among the 3 groups on POD7. Scale bar: 20 μm. (**C**) Quantified infiltrated CD68^+^ macrophages and M2-like (CD68^+^ and Arg1^+^) macrophages among the three groups (*n* = 6). (**D**) CD68 and iNOS staining CD68 in the flap among the 3 groups on POD7. Scale bar: 20 μm. (**E**) Quantified infiltrated CD68^+^ macrophages and M1-like (CD68^+^ and iNOS^+^) macrophages among the three groups (*n* = 6). SEM error bars are used. Significance (*): p value < 0.05; equal variances ANOVA with LSD post hoc analysis or unequal variances Dunnett’s T3 technique

Next, we investigated the influence of ABs on macrophage polarization in *vivo*. DiI-labeled ABs were injected in skin flap during surgery, and on POD3, CD68-positive macrophages internalized ABs **(**Fig. 7A**)**. Then, the polarization of macrophages was observed in the flap on POD7, identifying M1-like (CD68^+^ and iNOS^+^) **(**Fig. 7B**)** and M2-like (CD68^+^ and Arg1^+^) **(**Fig. 7C**)** macrophages. The ABs group showed an impressive decrease in M1-like macrophage expression as compared to the PBS group but no change in CD68^+^ macrophage expression **(**Fig. 7D**)**. In contrast, M2-like macrophages were markedly increased in the ABs group. Furthermore, 3D-ABs exhibited superior ability in promoting M1-to-M2 macrophage polarization compared to 2D-ABs **(**Fig. 7E**)**. In conclusion, better effects were observed in promoting the M2 polarization of macrophages by 3D-ABs.

### Differential expression of microRNAs in ABs produced by mADSCs in 3D culture

**Fig. 8:**
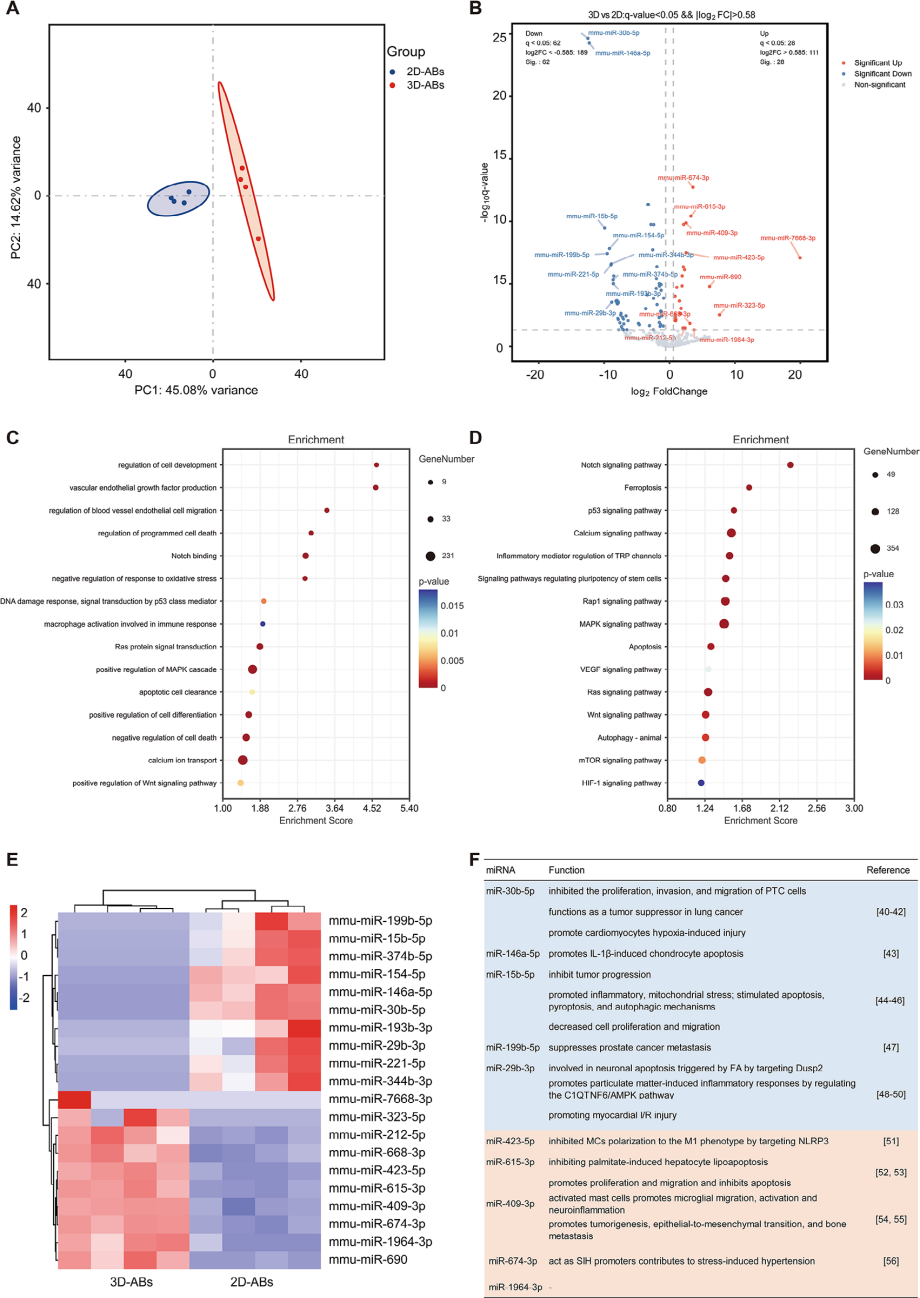
Differential miRNA profiles of ABs were produced by 3D cultivation of mADSCs. (**A**) Principal component analysis (PCA) of the miRNAs in 2D- and 3D-ABs was carried out. (**B**) To show which miRNAs were differently expressed, a volcano plot was created. (**C**) Target genes linked to biological processes that were inferred from differentially expressed miRNAs in 3D-ABs underwent GO analysis. (**D**) Target genes identified from differentially expressed miRNAs in 3D-ABs were analyzed using the KEGG. (**E**) In comparison to 2D-ABs, the top 10 miRNAs that were up- and down-regulated in 3D-ABs were plotted on a heatmap. (*n* = 4) (**F**) Compared to 2D-ABs, the biological roles of the top 10 up- and down-regulated miRNAs in 3D-ABs were investigated

To investigate disparities between 3D-ABs generated by 3D-cultured organotypic-like mADSCs and 2D-ABs, as well as the mechanisms underlying their therapeutic impact on ischaemic flaps, we performed miRNA sequencing analyses on both 2D-ABs and 3D-ABs. Principal Component Analysis (PCA) result revealed significant disparities in miRNA expression profiles between 2D-ABs and 3D-ABs, while samples within each group demonstrated strong reproducibility **(**Fig. 8A**)**. In comparison to 2D-ABs, 3D-ABs exhibited differential upregulation in a total of 28 miRNAs and differential downregulation in 62 miRNAs **(**Fig. 8B**)**.

We performed Gene Ontology Analysis (GO) and Kyoto Encyclopedia of Genes and Genomes Analysis (KEGG) analyses on the target genes of differentially expressed miRNAs in order to ascertain the functions of the miRNAs. GO analysis indicated enrichment associated with the promotion of ischaemic flap survival, including regulation of cell development, vascular endothelial cell proliferation and migration, regulation of programmed cell death, promotion of cell differentiation, inhibition of oxidative stress, apoptotic cell clearance, macrophage activation, DNA damage response via p53, calcium ion transport, Ras protein signal transduction, MAPK signaling pathway, positive regulation of Wnt signaling pathway, etc. **(**Fig. 8C**)** KEGG analysis also identified several signaling pathways associated with the promotion of ischaemic flap survival, including p53 signaling pathway, Calcium signaling pathway, VEGF signaling pathway, MAPK signaling pathway, Ras signaling pathway, Wnt signaling pathway, mTOR signaling pathway and HIF-1 signaling pathway, etc. In addition to the above pathways, KEGG analysis also found relevant indicators that affect flap survival, including inflammatory regulation, ferroptosis, apoptosis, and autophagy **(**Fig. 8D**)**.

We found that the top 10 downregulated and top 10 upregulated miRNAs in 3D-ABs were different from those in 2D-ABs **(**Fig. 8B **and E)**. The top 10 upregulated ones encompassed miR-7668-3p, miR-323-5p, miR-212-5p, miR-668-3p, miR-423-5p, miR-615-3p, miR-409-3p, miR-674-3p, miR-1964-3p and miR-690, while the 10 most markedly downregulated ones feature miR-199b-5p, miR-15b-5p, miR-374b-5p, miR-154-5p, miR-146a-5p, miR-30b-5p, miR-193b-3p, miR-29b-3p, miR-221-5p and miR-344b-3p **(**Fig. 8E**)**. The functions of these miRNAs have been documented in the literature [45–61]. Among them, the top five with the most notable expression in upregulation and downregulation havd been shown to contribute to the promotion of ischaemic flap survival **(**Fig. 8F**)**. In contrast to 2D-ABs, miR-423-5p exhibited the most pronounced upregulation in 3D-ABs. Elevated levels of miR-423-5p have the capacity to suppress macrophage polarization towards the M1 phenotype by targeting NLRP3, consequently dampening the inflammatory response [56]. Furthermore, miR-30b-5p standed out as the most significantly downregulated miRNA within the context of 3D-ABs. It has been demonstrated that increased miR-30b-5p levels inhibit the proliferation, invasion, and migration of PTC cells [45], function as a tumor suppressor in lung cancer [46], and promote cardiomyocytes hypoxia-induced injury [47].

Collectively, these findings suggest that 3D-ABs possesses a distinct miRNA profile that fosters cell proliferation, suppresses cell death, mitigates hypoxic injury, and dampens inflammatory responses. Together, these effects contribute to the enhanced survival of ischaemic skin flaps.

## Discussion

ADSCs demonstrate pluripotency, capable of differentiating into various mesenchymal tissue lineages, including fat, cartilage, muscle, and bone [62]. Given their rapid proliferation and ample presence in the body, ADSCs hold significant therapeutic promise for tissue repair [63]. Consequently, ADSCs-ABs offer substantial therapeutic potential in tissue repair. It has been found that ABs transport proteins, miRNAs, and other nucleic acids to recipient cells in order to control a variety of biological processes [64]. Various physiological or pathological conditions significantly influence the functional composition of ABs, indicating that alterations in the microenvironment of parent cells can impact ABs cargo [25]. To date, no reports have explored the influence of 3D culture on ABs’ functionality. The primary discovery of this study is that ABs generated by mADSCs in 3D cultures effectively enhance the survival of ischaemic flaps through the promotion of angiogenesis, inhibition of apoptosis and necrosis, reduction in oxidative stress, and the induction of macrophage M2 polarization.

Growing data in recent years has suggested that cells exhibit different behavior when cultured in 3D compared to traditional 2D culture [65]. Three-dimension culture can enhance cellular physiology by promoting greater interactions between cells and their surrounding matrix [66]. Through mechanisms including cell-matrix adhesion and dynamic feedback, the physical and mechanical characteristics of the extracellular environment are crucial in controlling a number of cellular activities in ADSCs, including adhesion, proliferation, migration, and differentiation [67]. Due to the significant differences in growth pathways between planar 2D substrates and 3D suspended cultures, the contact state undergoes a marked change. Suspension culture is achieved by allowing cells to self-aggregate into spherical structures, in which they are more closely resembling actual tissue states [68].

This study investigated the effects of 2D and 3D-ABs on ischaemic flap survival. The effects of ABs on ischaemic endothelial cell rescue and macrophage polarization were investigated in *vitro*, while in *vivo* experiments were performed to examine the impact of ABs on ischaemic flap survival and macrophage polarization. The results suggest that ABs hold significant promise for treating ischemic injury, with 3D-ABs, being more biologically pertinent, demonstrating superior therapeutic effects, especially in treating ischemic flaps. The promotion of angiogenesis, inhibition of oxidative stress, suppression of apoptosis, and alleviation of inflammatory damage are critical for flap survival, and our experimental results demonstrate that ABs play a significant role in these aspects, with 3D-ABs displaying superior therapeutic effects.

To further investigate the potential mechanisms underlying the promotion of ischemic flap survival by 3D-ABs, we performed miRNA sequencing. The miRNA profile of 2D-ABs supports factors conducive to ischaemic flap survival, including angiogenesis promotion, apoptosis inhibition, oxidative stress mitigation, and macrophage M2 polarization induction. In contrast, 3D-ABs exhibit significantly heightened anti-inflammatory characteristics, reinforced promotion of cell survival, facilitation of angiogenesis, and marked reduction in oxidative stress. Specifically, certain miRNAs, such as miR-423-5p, miR-615-3p, and miR-409-3p, exhibit decreased expression, while those associated with pro-inflammatory responses, cell death, and growth inhibition, like miR-30b-5p, miR-146a-5p, and miR-15b-5p, are expressed at lower levels. This differential expression contributes to the enhanced ability of 3D-ABs to foster ischaemic flap survival.

We evaluated the role of 3D-ABs in promoting survival of ischaemic flaps through a series of tests. While the control group did not show as much flap survival in the animals treated with ABs, the mice treated with 3D-ABs performed better than the mice treated with 2D-ABs. Additionally, there are various methods for administering EVs in animal models. In this study, we used an in-situ delivery method within the flap during surgery, which allows for high local concentrations of ABs, reducing the loss of ABs in circulation and potential adverse effects in other organs. It has been suggested that administered EVs may also distribute to the lungs and spleen, which may influence brain inflammation events. Further optimization of the delivery method for ABs therapy in ischaemic flaps is necessary, taking into account factors such as dosage, treatment time, stability during in *vivo* circulation, and efficiency in reaching target cells via the vasculature.

At the same time, although our study addresses several key aspects of ABs-based ischemic flap therapy, there are potential limitations in our understanding of underlying mechanisms, optimal dosing regimens, and long-term effects. In this study, we did not discuss and verify the role of a single miRNA in *vivo* in depth. In subsequent studies, we will conduct specific verification on the role of a single miRNA in ischemic skin flaps. Moreover, we used 0.5 mg/ml concentration of 2D and 3D-ABs for comparison, but in Fig. 5B-D both 1- and 1.5-2D-ABs showed better therapeutic effects than 0.5-2D-ABs. Does this mean that higher concentrations of 3D-ABs can break through the therapeutic limit in ischeamic flap? Or will high concentrations of ABs bring their own apoptotic factors to cause cell death? Therefore, the next exploration needs to determine the ABs dosing regimen to determine the optimal dose and frequency to maximize the therapeutic effect while minimizing potential side effects. This may involve dose-response studies to determine the most effective dose range. Furthermore, it is necessary to conduct a long-term follow-up study, in this study only the ischeamic flap quality of POD7 was discussed to evaluate the durability and sustainability of the therapeutic effect of ABs on ischeamic flap survival. This will provide valuable insights into the persistence of treatment effects over time. Finally, consider initiating clinical trials to evaluate the fact that ABs can be derived from autologous cells, and their biosafety is relatively reliable, and there have been clinical studies using autologous ADSCs for implantation treatment, which has important guiding significance for the clinical transformation of 3D-ABs [20].

The effects of 3D-ABs on angiogenesis, reduction of oxidative stress, and macrophage polarization have a direct impact on improving clinical outcomes: first, the pro-angiogenic ability of 3D-ABs is crucial for restoring adequate blood supply to ischemic tissues. By stimulating angiogenesis, 3D-ABs can promote tissue repair and regeneration, ultimately improving clinical outcomes in patients with ischemic injury; Second, oxidative stress plays a central role in the pathogenesis of ischemic diseases, exacerbating tissue damage and impairing recovery. The reduction of oxidative stress by 3D-ABs is crucial for attenuating tissue damage and inflammation associated with ischemic injury; Furthermore, the ability of 3D-ABs to polarize macrophages toward the M2 phenotype suggests their potential to modulate inflammatory responses and promote tissue healing from ischemic injury. Taken together, the observed effects of 3D-ABs have profound implications for the treatment of ischemic injury in the clinic. But this also involves more challenges, such as using a safe concentration and dosage, determining the administration method and toxic and side effects. And in a larger direction, in further human experiments, whether 3D-ABs can be used for universal treatment, whether there will be off-target effects of miRNA and further packaging into biological materials to improve the therapeutic effect. ABs derived from autologous cells do not cause immune rejection when used on themselves, but this greatly limits clinical application. Therefore, research on removing immunogenic ABs is very important. It is true that the miRNA profile in ABs is relatively complex, and it is inevitable that there will be damaging miRNAs, which will bring unknown toxic and side effects. Determining and unifying the contents can speed up the process of clinical application. At the same time, ABs can also be used as raw materials for further packaging and processing to reduce their complexity, such as making nanovesicles and replacing their contents, which can reduce immune responses and reduce toxic side effects. This needs further exploration in future research.

## Conclusion

In conclusion, this research offers a valuable resource for harnessing ABs derived from 3D-cultured pheroids mADSCs to enhance the survival rate of ischaemic skin flaps. ABs extracted from 3D cultured ADSCs have stronger biological therapeutic capabilities. Mechanistically, they stimulate angiogenesis, reduce oxidative stress, inhibit apoptosis, and enhance the shift in macrophage polarization from M1 to M2. This study provides valuable resources for using 3D-AB to improve the survival rate of ischemic skin flaps.

## Methods

### Cells culture

Mouse ADSCs (MUBMD-01001) were obtained from Ori Cell Bio Co., Ltd. The mADSCs was cultured in mADSC complete culture medium (Ori Cell Bio, MUXMD-90,011). mADSCs were cultivated at 37 °C in an incubator with 5% CO2 and 95% air. The studies were conducted using mADSCs during the fourth passage.

Raw264.7 (CL-0190) and HUVECs (CL-0122) were obtained from Procell Life Science & Technology Co., Ltd. We cultured the cells in an incubator in 5% CO_2_ and 95% air at 37 °C. DMEM (Gibco, C11995500BT) supplemented with sterile 10% FBS (Gibco, 10,099,141 C) and 1% penicillin‒streptomycin (Gibco, 1,719,675) was used to culture the cells.

Add the cell suspension (100 µl/well, 1000 cells/well) to the 96U-shaped well plate (Engineering For Life, EFL-SP101) of the cell pellet treated with the anti-adhesion coating solution. Spherical 3D-mADSCs were formed after 48 h of culture in the plate, and the next experiment was carried out. Optical microscope was used to observe the status of 2D-mADSC and 3D-mADSC. The cytoskeleton was labeled with phalloidin-FITC (Actin-Tracker Green; Beyotime, C1033), and the nucleus was stained with DAPI (Abcam, ab228549). The Calcein/PI cell viability and cytotoxicity detection kit (C2015S) were employed, following the manufacturer’s instructions, to stain 2D- and 3D-mADSCs, for the purpose of observing cell viability. And the cell morphology of 2D- and 3D-mADSCs was observed under a confocal microscope.

### Animals

The Wenzhou Medical University Animal Welfare and Use Committee approved each animal test that was carried out in accordance with the China National Institutes of Health’s Guidelines for the Welfare and Use of Lab Animals (wydw2024-0057). Male C57BL/6 mice (mean body weight 20–30 g, 6–8 weeks) were provided by the Wenzhou Medical University Experiment Animal Center (no. SCXK [ZJ] 2015–0001). All mice were kept under normal conditions (21–25 °C, humidity: 50–60%, 12-h light/dark period) and possessed free food and beverages. Every mouse utilized in this research has a background in C57BL/6J.

### Isolation and characterization of ABs

After being treated with 0.5 µmol/L STS (Med Chem Express, HY-15,141) for apoptosis induction, 2D or 3D-mADSCs were incubated at 37 °C in 5% CO_2_. Cell supernatants were collected after 12 h and centrifuged for 5 min at 300 × g to eliminate any remaining cell debris. After that, the supernatants were centrifuged three times for 30 min at 2,000 × g. ABs derived from 2D or 3D-mADSCs were then resuspended in PBS (Procell, PB180327) for further use. To ascertain the protein composition of 2D or 3D-ABs, we employed the BCA protein assay. Utilize a scanning electron microscope (SEM) to examine the morphology and estimate the size range of the isolated ABs. The accurate size distribution of 2D or 3D-ABs (gated size with mouse platelets) was measured using flow cytometry. Western blotting (WB) experiments was conducted to identify ABs using surface marker proteins (H3(Protein Technology Group, 17168-1-AP), H2B (ABclonal, A1958), C1QC (ABclonal, A9227) and C3B (ABclonal, A13283)). The WB experimental method used here is as previously described in a referenced article [12]. β-Actin (Abcam, ab213262) was employed as a quantitative indicator in WB. In accordance with the manufacturer’s instructions, 2D- and 3D- ABs were stained with the Annexin V-FITC/PI Cell Apoptosis Detection Kit (Servicebio, G1511) to identify unique PS signs on the ABs. FCM was employed to evaluate the purity of the ABs.

### Internalization of ABs into HUVECs and Raw264.7 in *vitro*

After plating the HUVECs and Raw264.7 onto plates, they were kept at 37 °C for the whole night. 2D or 3D-ABs were pre-labeled with the Cell Plasma Membrane Staining Kit with DiI (Beyotime, C1991S) as directed by the manufacturer and centrifuged three times at 2,000 × g for 30 min in PBS. DiI 2D or 3D-ABs were then co-cultured with HUVECs for 12 h at a concentration of 10 µg/ml. After the cell nuclei were fixed for 15 min at 4 °C with 4% paraformaldehyde (Solarbio, P1110), they were counterstained with Hoechst 33,342 (Biosharp Life sciences, BL803A). Cell morphology of HUVECs and Raw264.7 with DiI-ABs (2D and 3D) was observed under a confocal microscope.

### Hypoxic cell model

Cells were chosen from a healthy logarithmic growth phase, trypsined with 0.25% (Gibco, 25,200,072), followed by centrifugation, supernatant removal, resuspension, and subsequent seeding in cell culture plates for the respective experiments. The normoxic group was cultured in a standard cell culture incubator. The hypoxia group and drug administration group were incubated in a humidified hypoxic chamber at 37 °C, with an atmosphere (1% O_2_, 5% CO_2_, and 94% N_2_) for 24 h.

### Cell counting kit 8

HUVECs were plated in 96-well plates at a density of 5 × 10^3^ cells per well, resulting in 50% cell confluence. The cells were then exposed to a variety of therapies, including PBS, 2D-ABs at different concentrations (ranging from 0 to 70 µg/ml), and a 10 µg/ml concentration of 2D/3D-ABs. The cells were co-incubated with gradient ABs concentrations in 96-well plates within a hypoxic incubator. In contrast, NC group cells were cultured in 96-well plates in a normoxic incubator, following the procedure outlined in the “Hypoxic cell model”. Subsequently, 10 µL of CCK-8 solution (Med Chem Express, HY-K0301) was introduced to the wells, and the cells were incubated at 37 °C for 3 h. A microplate reader was used to measure absorbance at 450 nm.

### Tube formation assay

On ibidi µ-slides (Ibidi, 81,506) covered with 10 µL/well of growth factor-reduced Matrigel (Corning, 356,234), the in *vitro* angiogenic activity of HUVECs was evaluated. HUVECs were reseeded in the prepared ibidi µ-slides after being stained for 30 min with the cell-permeable dye (calcein AM; Beyotime, C2012). Using a confocal microscope, capillary-like tube development was seen during an 8-hour incubation period at 37 °C in a cell culture incubator. These structures were defined as tubes with a length four times their width. Tube lengths were quantified in duplicate wells, and the average length was calculated using ImageJ software.

### Transwell assay

Polycarbonate membrane Transwell inserts (8.0-µm) were used in cell migration tests to evaluate the in *vitro* migratory ability of HUVECs in each group (Corning, 3422). HUVECs were positioned in the top chambers and cultured at 37 °C for eight hours following the prescribed procedures. Subsequently, crystal violet staining and 4% paraformaldehyde fixation were applied to each chamber’s cells. A computerized microscope was used to take pictures of the migrated cells.

### Apoptosis detection

The Annexin V-FITC/PI Cell Apoptosis Detection Kit was used to stain the HUVECs in each group in compliance with the guidelines given. The apoptosis levels of HUVECs were subsequently detected using flow cytometry.

### Immunocytochemistry

HUVECs in each group were subjected to fixation for 30 min with 4% paraformaldehyde. Subsequently, they underwent a 5-minute permeabilization step using 0.1% Triton X-100 (Aladdin, T109027) in PBS, followed by a 30-minute blocking procedure with 10% goat serum (Beyotime, C0265) in PBS. Intracellular ROS levels in HUVECs were assessed using DHE (Dihydroethidium; Beyotime Biotechnology, S0063) following the manufacturer’s protocol. Dead cell levels in HUVECs were determined through TUNEL (In Situ Cell Death Detection Kit, Fluorescein; Roche, 11,684,795,910) according to the manufacturer’s instructions. JC-1 (Beyotime Biotechnology, C2003S) was used to measure the mitochondrial membrane potential in HUVECs as per the manufacturer’s guidelines.

### Macrophage polarization assay

Raw264.7 cells from different stimulation groups were trypsined, resuspended, and chilled at 4 °C, followed by fixation in 70% alcohol for 2 h. After two resuspensions in PBS and adjustment to a concentration of 2 × 10^6^ cells per EP tube, antibodies were developed in the cells: Arg1 (1:50; Cell signaling Technology, 93,668 S) and iNOS (1:50; Cell signaling Technology, 13,120 S) for 30 min. Subsequently, they were resuspended twice in PBS, treated with secondary antibodies (goat anti-rabbit IgG - H&L DyLight® 488 (Abcam, ab96883); goat anti-mouse IgG - H&L DyLight® 594 (Abcam, ab96873)), and then washed twice with PBS prior to conducting flow cytometry analysis.\ **qPCR**.

Using the mirVana miRNA Isolation Kit (Ambion) and the manufacturer’s instructions, total RNA was extracted. Total RNA was quantified using the Nanodrop 2000 (Thermo Fisher Scientific Inc., USA). The Agilent 2100 Bioanalyzer (Agilent Technology, USA) was utilized to evaluate the integrity of RNA.

Quantitation was completed via a two-step reaction: reverse transcription (RT) and PCR. Every RT process involved 0.5 µg of RNA, 2 µL of 5 × TransScript All-in-One SuperMix for qPCR and 0.5 µL of gDNA Remover (10 µL). The reaction was performed using a GeneAmp® PCR System 9700 (Applied Biosystems, USA) for 15 min at 42 °C and then for 5 s at 85 °C. Subsequently, the 10 µL RT reaction mixture was desaturated 10 times at -20 °C in nuclease-free water. Real-time PCR was performed using a Light Cycler® 480 II real-time PCR instrument (Roche, Switzerland) with 10 µL of PCR mix, 1 µL of cDNA, 5 µL of 2 × PerfectStart Green qPCR SuperMix (TransGen Biotech Co., AQ601), 0.2 µL of forward primer, 0.2 µL of reverse primer and 3.6 µL of nuclease-free water. The reaction was performed in a 384-well optic plate for 0.5 min (Roche, 04729749001) at 94 °C and then for 45 cycles of 5 s at 94 °C and 30 s at 60 °C. The specimens were analysed three times. After the PCR cycles were complete, a melting curve assay was used to verify the production of the anticipated PCR products. The following primer sequences were synthesized by GeneChem using the mRNA sequences acquired from the NCBI database: *Tnf* 5’- *GATCGGTCCCCAAAGGGATG* − 3’ (forward) and 5’- *CCACTTGGTGGTTTGTGAGTG* − 3’ (reverse); *Nos2* 5’- *TCTAGTGAAGCAAAGCCCAACA* − 3’ (forward) and 5’- *CCTCACATACTGTGGACGGG* − 3’ (reverse); *Il6* 5’- *CCTTCTCCACAAGCGCCTTC* − 3’ (forward) and 5’- *GGAAGGCAGCAGGCAACA* − 3’ (reverse); *Cd163* 5’- *GTGCTGGATCTCCTGGTTGT* − 3’ (forward) and 5’- *CGTTAGTGACAGCAGAGGCA* − 3’ (reverse); *Arg1* 5’- *GTAGACCCTGGGGAACACTAT* − 3’ (forward) and 5’- *ATCACCTTGCCAATCCCCAG* − 3’ (reverse); *Il10* 5’- *GCTGTCATCGATTTCTCCCCT* − 3’ (forward) and 5’- *GACACCTTGGTCTTGGAGCTTAT* − 3’ (reverse); *Actb* 5’- *CTACCTCATGAAGATCCTCACCGA* − 3’ (forward) and 5’- *TTCTCCTTAATGTCACGCACGATT* − 3’ (reverse). We normalized the expression of the target mRNAs to *Actb* mRNA expression, respectively. The 2-ΔΔCt method was used for qPCR analyses.

### Western blotting

Using cold RIPA lysis buffer (Beyotime, P0013B) enhanced with phenylmethanesulfonyl fluoride (PMSF; Beyotime, ST506) as well as a protease and phosphatase inhibitor cocktail (Beyotime, P1046), Raw364.7 cell samples were homogenized. To obtain cell lysate, the homogenates were centrifuged at 20,000 g for 30 min at 4 °C. The Omni-EasyTM Instant BCA Protein Assay Kit was used to measure the protein concentrations.After loading 30 mcg of protein onto 4–22% SDS–PAGE gels, the protein was subsequently moved into PVDF membranes (Millipore). When primary antibodies were applied, the PVDF membranes had been diluted using 5% skim milk (BD Biosciences, 232,100) and incubated at 4 °C for 15 h. The membranes followed by treatment with HRP-conjugated secondary antibodies at room temperature for 1.5 h. An Omni-ECL Pico Light Chemiluminescence Kit (EpiZyme, SQ201) was utilized to identify protein bands, as well as a ChemiDoc system (Bio-Rad) was employed to display the results. Using Image Lab software from Bio-Rad, bands were analyzed. The following proteins were targeted by the main antibodies (1:1,000) in the study: iNOS (Cell Signaling Technology, cat# 13,120 S), Arg1 (Cell Signaling Technology, cat# 93,668 S) and β-actin (Abcam, cat# ab213262).

### Random-pattern skin flap model

Mouse random-pattern skin flap model was done as previously described [12]. The C57BL/6 mice were anesthetized via intraperitoneal injection with a 1% (w/v) solution of sodium pentobarbital. Afterward, using an electric shaver and depilatory cream, the fur from the back of the anesthetized model mouse with a randomly-pattern flap was shaved. Under sterile conditions, sterile instruments were used to lift the caudal skin/sarcoma flap (dimensions: 1.5 × 4.5 cm^2^) under the dorsal fascia of the mouse. Subsequently, the sacral arteries that provided blood supply to the flap were surgically excised, including both the left and right sides. The mice in the PBS group, 2D-ABs group, and 3D-ABs group received subcutaneous injections of 100 µl (administered at 8 injection sites) containing PBS and ABs (at concentrations of 0.5, 1, or 1.5 mg/ml) using a microinjection needle along the immediate extension axis. At last, 4 − 0 nonabsorbable silk sutures were used to sew the split flap straight into the donor bed. To prevent postoperative infection, eliminate the odor of blood from the wound, and deter any biting, the wound was disinfected with 1% iodophor twice daily following the surgery.

We randomly classified the C57BL/6J mice into 7 treatment groups (mg/ml): the PBS (*n* = 6), 0.5-2DABs (*n* = 6), 1-2DABs (*n* = 6), 1.5-2DABs (*n* = 6), 0.5-3DABs (*n* = 6), DiI-2D-ABs (*n* = 5) and DiI-3D-ABs (*n* = 5) groups. All mice were tagged with ear tags and placed randomly, with five mice per cage, maintained under suitable temperature and humidity conditions with access to ample food and water. The skin flap was evenly divided into three zones, from proximal to distal, namely zone I, zone II, and zone III. Zone II is where our subsequent experimental was performed.

### Internalization of ABs into ECs and microphages in *vivo*

Following the method mentioned above, 0.5 mg/ml DiI-ABs were employed for in situ injection into the skin flap on POD3, a zone II skin flap was obtained for frozen section staining. ECs and macrophages were identified using CD31 (Servicebio, GB11063-2-100) and CD68(Santa Cruz Biotechnology, sc-20,060), respectively. Primary antibodies were left to incubate overnight in a refrigerator set at 4 °C. The subsequent day, after being washed thrice with PBS, secondary antibodies (goat anti-rabbit IgG - H&L DyLight® 488; goat anti-mouse IgG - H&L DyLight® 594) were added, and the samples were incubated for one hour at 37 °C in a water bath. DAPI was used to stain the cell nuclei, and the observation of ABs’ phagocytosis by endothelial cells and macrophages within the flap was conducted using a confocal microscope.

### Infrared thermal imaging scan

Thermal images of the ischaemic flaps were captured using the FLIR One Pro (FLIR Systems, Inc. USA) external probe for infrared thermal imaging via a mobile phone. Average temperatures of the operating area and the head and neck area were measured independently (normal skin). Delta-T, defined as the temperature difference between the operating area and normal skin, was determined. Temperature differences between the groups were analyzed and compared. The basal body temperatures of mice in each group remained within the normal range. A closer approach to a delta-T of 0 °C indicated better flap recovery.

### Laser doppler blood flow (LBDF)

The vascular network of the flap was visualized using LDBF analysis. After anesthesia on POD7, the mouse was maintained in a disturbance-free environment. Subsequently, a laser Doppler instrument was employed to evaluate the skin flap’s blood supply. The LDBF analysis was conducted following established procedures. The moorLDI Review software (ver. 6.1; Moor Instruments) was utilized to calculate perfusion units (PUs) for the assessment of blood flow. Three measurements of each mouse’s blood flow were made, and statistical analysis was performed on the average result.

### Immunohistochemistry

The mouse flap tissue in zone II was fixed using 4% paraformaldehyde. After paraffin embedding, the flap tissue from area II was sectioned into 4-µm sections. In every IF experiment, xylene was used to deparaffinize the sections. After the tissue had been deparaffinized, it was rehydrated and put through a sodium citrate buffer antigen retrieval procedure. Following this, in PBS containing 0.1% Triton X-100, 10% goat serum was used to block the sections. After that, they were incubated for one hour the next day at room temperature with secondary antibodies and throughout the entire night with primary antibodies at 4 °C. DAPI was used to stain the cell nuclei. The primary antibodies utilized were specific to CD31 (1:200), CD68 (1:200), α-SMA (1:200; Proteintech, 67735-1-Ig), iNOS (1:200), and Arg1 (1:200). Goat polyclonal secondary antibody against rabbit IgG - H&L DyLight-488, goat anti-mouse IgG - H&L DyLight-488 (Abcam, ab96871), goat anti-rabbit IgG - H&L DyLight-594 (Abcam, ab96885), and goat anti-mouse IgG - H&L DyLight-594 were among the secondary antibodies.

For TUNEL assays on frozen skin sections, we used an in situ cell death detection kit according to the manufacturer’s instructions. Dihydroethidium (DHE) staining was performed on frozen skin sections as per the manufacturer’s protocol to detect collagen damage. F-CHP (3Helix Inc., FLU300) was applied according to the manufacturer’s protocol.

### RNA isolation and library preparation

Total RNA was extracted as previously described. In order to generate small RNA libraries, 1 µg of total RNA from every sample was prepared using the NEBNext Small RNA Library Prep Set for Illumina kit (Cat. No. NEB#E7330S, NEB, USA) in accordance with the instructions provided by the manufacturer. To summarize, both ends of the total RNA were ligated to adapters, and then reverse transcription to cDNA and PCR amplification were performed. Small RNA libraries were produced by isolating and purifying PCR products with a bp range of 140–160. The Agilent Bioanalyzer 2100 system was used to assess the quality of the library.

The Illumina Novaseq 6000 platform was used for sequencing, producing 150 bp paired-end reads. OE Biotech Co., Ltd. conducted small RNA sequencing and analysis (Shanghai, China).

### MiR-seq

Base calling was used on the original readings in order to produce sequence data, also known as raw data/reads. Subsequently, low-quality readings were filtered out and reads containing poly (A) and 5’ primer contamination were removed. To acquire clean reads, further filtering was performed on reads from the raw data that did not include a 3’ adapter, insert tag, or that were longer than 41 nt or less than 15 nt. The length distribution of the clean sequences in the reference genome was determined, then the sequences were aligned and subjected to the Bowtie [69] search against Rfam v.10.1 (http://www.sanger.ac.uk/software/Rfam) [70], rRNA, scRNA, Cis-reg, snRNA, tRNA and other RNAs were annotated and filtered. Then, cDNA sequence and Repbase [71] database of species repeat sequence were also identified with Bowtie software. The mature miRNAs were identified by aligning against miRBase v22 database (http://www.mirbase.org/) [72], and the expression patterns in different samples were analyzed.

miRNAs that were differentially expressed were determined and filtered using a threshold of FC > 2 and q value < 0.05. For experiments with biological replicates, the DEG method in the R package was used to determine the q value; for experiments without biological replicates, the Audic Claverie statistic was used. The targets of differentially expressed miRNAs were predicted by using software miranda in animal, with the parameter as follows: S ≥ 150, ΔG ≤ − 30 kcal/mol and demand strict 5’ seed pairing.

R was utilized to conduct GO enrichment and KEGG pathway enrichment analysis of distinct expressed miRNA-target genes, respectively, utilizing the hypergeometric distribution.

### Statistics

Statistical assays were completed via the SPSS 22 programme (USA). All data are described as the average ± SEM. All of the data displayed here have undergone normalization to account for unintended sources of variance. To find differences between 3, 4, or 5 groups, an ANOVA was used, followed by LSD (equal variances assumed) post hoc analyses or Dunnett’s T3 (equal variances not assumed). The study employed independent-specimen t tests to ascertain group differences. *P* < 0.05 indicated statistical significance.

## Data Availability

Additional data collected during this study are available from the corresponding author upon reasonable request.
